# The Effectiveness of Leadership Interventions on Cardiologists’ Performance Using Benchmarking as a Tool

**DOI:** 10.7759/cureus.66744

**Published:** 2024-08-13

**Authors:** Abdulrahman almesned, Yasser A Bhat

**Affiliations:** 1 Pediatric Cardiology, Prince Sultan Cardiac Center Qassim, Buraidah, SAU

**Keywords:** cost reduction, attitude of health personnel, safety study, cath lab, health care leadership

## Abstract

Background: Aligned with the Health Sector Transformation Strategy of Saudi Vision 2030, the study analyzed the attitudes and behaviors of cardiologists toward change and identified factors that could either facilitate or hinder the success of leadership interventions. At our cardiac center, the cath lab department is at the forefront of operations, accounting for 80% of the procedures. Our team members may not be fully equipped with the necessary attitudes and behaviors to drive successful improvement projects. Therefore, our top priority is ensuring they remain productive and engaged throughout the process. This is especially crucial because 60% of our budget is allocated to the cath lab department.

Objectives: The study aimed to assess the effectiveness of a leadership intervention on cardiologists' performance in terms of safety, speed, and cost. The research analyzed the behavior and attitude of cardiologists towards change and encouraged progress and collaborative learning between doctors, using benchmarking as a tool. Besides, the study sought to determine the contribution of the interventions used to overall efficiency in performing interventions. This case study focuses on four main aspects of the program. First, it aims to explore an innovative approach to improving the PSCCQ cath lab for patients. Second, it assesses the collective effort of all participants involved in the program. Third, it analyzes the program results and compares them with those of international experiences. And finally, it examines the program's potential benefits for our patients.

Methods: The study's objectives were evaluated through qualitative analysis of in-depth interviews and quantitative data analysis of three variables in the cath lab: radiation dose, time, and inventory. The reason for using mixed methods was to comprehensively understand the same concept from different angles.

Results: According to the study, participants improved the safety and effectiveness of our cath lab by reducing the radiation dose and its cost. The study revealed a 52% decrease in the radiation dose for diagnostic cases and an 11% decrease for interventional cases. Similarly, the cost of the radiation dose decreased by 28% for diagnostic cases and 11% for interventional cases. During the observation, it was noted that the participants were highly engaged and willing to adapt to the situation. Some even viewed it as an opportunity for personal growth and improvement in the cath lab. However, they stressed the significance of awareness as a crucial element in improving their behavior and reinforcing it as the foundation for maintaining progress. Furthermore, the study revealed that collaborative work among the participants could have been more optimal.

Conclusion: The study concludes that implementing innovative improvements to the cath lab was a necessary yet complex undertaking. Participants were more inclined to embrace the changes when they were easily understandable and motivating. The study recommends the appointment of a change agent, the establishment of benchmarks, and the creation of a collaborative working environment between leaders and staff. Above all, the leader should support and sponsor the change to facilitate the transition at various levels.

## Introduction

The Kingdom of Saudi Arabia's healthcare industry has the third largest share of national expenditures, 16.4% of the 2020 budget. The Kingdom of Saudi Arabia's population is expected to increase from 33.5 million in 2018 to 39.5 million by mid-2030. Furthermore, the number of elderly aged between 60 and 79 is expected to rise from 1.96 million in 2018 to 4.63 million by mid-2030 [1]. In recent years, the Ministry of Health (MOH) has proposed new reforms that align with the National Transformation Program 2020 and the Saudi Vision 2030. These reforms aim to institutionalize the health system and improve the financing system to increase efficiency and effectiveness in service delivery [2]. Cardiac disease is the Kingdom of Saudi Arabia's most significant cause of mortality. Thus, in partnership with the Vision 2030 Realization Office of the MOH, Saudi Arabia's cardiac community published a comprehensive national cardiac plan for the Kingdom of Saudi Arabia in 2019. The cornerstone of this approach is providing effective and high-quality care based on the patient's interests [3,4]. MOH's Prince Sultan Cardiac Center Qassim (PSCCQ) is the only tertiary cardiac center in the province of Qassim that provides elective and emergency cardiac procedures. PSCCQ's core business is the cardiac catheterization laboratory (cath lab), where coronary angiography and stenting of diseased vessels are conducted regularly. In 2019, a significant proportion of procedures, amounting to over 80% of the total, were conducted in the cath lab of PSCCQ. This area of the facility accounted for approximately 60% of our budgetary expenditure. The primary challenge was to improve critical indicators, such as safety, cost-effectiveness, and efficiency, by implementing benchmarking strategies as a leadership intervention.

This study is closely aligned with the National and MOH 2030 Visions and the National Cardiac Strategy for the Kingdom of Saudi Arabia [5]. The research aimed to improve the efficacy and safety of cath lab procedures by analyzing cardiologists' behavior and attitudes toward adopting radiation dose benchmarking strategies, optimizing procedure time, and using inventory.

## Materials and methods

This study was conducted at PSCCQ's adult cath lab, the only cardiac center in the province receiving all adult cardiac cases. It aimed to benchmark methods to enhance cardiologists' work safety and cost efficiency by analyzing statistical data on their time usage, radiation exposure, and material costs. The study was also intended to highlight, understand, and extrapolate the effects of leadership by utilizing complex data and statistics to study the impact of cardiologists' behavior.

The interventional adult cardiologists at our center, despite their diversity in nationality, age, and training locations, are a limited group of six practitioners, leading the investigators to include all group members to increase the study's depth of insight. The sampling methodology used for this study was non-probability sampling through convenience sampling, as the sample was readily available and included all participants. Individually, in-depth interviews were conducted to determine the underlying thoughts, values, and attitudes behind participants’ behaviors and actions. The complexity of coronary procedures varied, so only diagnostic and interventional coronary interventions that required no more than one stent were included in the analysis to enable internal benchmarking. The research was conceived as a case study and aimed at evaluating the effect of leadership on cardiologists' efficiency (safety, speed, and cost). The objective of this study was to explore cardiologists' perspectives on change by employing qualitative analysis of in-depth interviews. Additionally, it aims to quantitatively analyze the impact of benchmarking on cardiologists' behaviors in the catheterization laboratory by examining three variables: radiation dose, procedure time, and inventory usage. Furthermore, the study seeks to gain insights into how physicians collaborate, learn from each other, and assess the influence of leadership through qualitative analysis of interviews.

Qualitative data from in-depth interviews with the cardiologists were analyzed to evaluate the effect of the benchmarking on the cardiologists' behavior in the cath lab. Quantitative data analysis of three variables in the cath lab (radiation exposure, time, and inventory) was obtained and compared with the available data before the research was initiated. For the qualitative data, an in-depth interview was conducted with the participating cardiologists.

Quantitative data on safety, speed, and cost were collected based on a literature review of cath laboratory benchmarks, as conducted by Christopoulos et al. [6]. The cases were categorized based on the procedure type, i.e., diagnosis or intervention. Three cath lab nurses were assigned to collect the data. The data collection took place over three months, from June to August 2020. Additionally, the data were collected retrospectively for the same variables before the study was initiated to evaluate if the leadership interventions impacted the research participants' results. The cath lab machine determines the safety variable by measuring the total air kerma (K) and the dose area product (DAP), as reported by Kuon et al. [7]. The time taken from the sheath-in to the sheath-out defined the speed [8], while the cost was calculated using the inventory MOH prices of 2020 in Saudi Arabian Riyals (SAR). The study focused on the percentage of cost reduction instead of the net cost of the individual procedure.

Analysis of the data

Quantitative data were analyzed using basic descriptive statistical techniques, which included calculating the mean and percentage of reduction/increase by using the inbuilt software in Google Spreadsheets. While the radiation dose was compared to published results, the speed and cost were not compared, as the authors needed help finding similar benchmarks in the literature. It should be noted that the study's primary objective was to assess the impact of the leadership intervention and not compare the findings with those of international cath labs. The framework by Braun and Clarke was implemented for qualitative data analysis, employing the Six-Phase Guide [9].

Ethical consideration

The ethical framework of the local health cluster was followed, ethical approval was obtained, and all participating cardiologists signed informed consent forms. The informed consent included written explanations of what this research was about for all participants, ensuring their understanding that participation was voluntary and they were free to leave at any point of the study. All participants provided benchmarking data and gave their informed consent. However, they were informed that the benchmarking data would not be considered in their evaluation.

## Results

Quantitative results

During phase II of the study, which took place from June to August 2020, 83 elective cases were included as they met the inclusion criteria. These cases were compared with the 39 elective cases from phase I (March-May 2020) of the cath lab log book. Of the 83 cases, 18 (21%) were diagnostic procedures, while 65 (79%) were interventional procedures. Table 1 presents a comparison of variables between phases I and II for diagnostic procedures.

**Table 1:** Displays phase I and phase II variables for diagnostic procedures K: air kerma; mGy: milligyres; DAP: dose area product; mGy/M^2^: milligyres per meter square

Parameter	Mean	
	Phase I, N=31	Phase II, N=18
Duration (min)	26.9	19.6
Fluoroscopy time (min)	8.6	5.8
Total K (mGy)	542.8	258.6
DAP (mGy/M^2^)	54.1	44.6

Seventy-three cases were included in interventional procedures. 89% of cases were in phase II, which showed a 15% reduction in procedure time and an 11% reduction in the overall radiation dose, while DAP increased by just 6%. Table 2 compares variables between phase I and phase II for interventional procedures.

**Table 2:** The parameters for phase 1 and phase II interventional procedures K: air kerma; mGy: milligyres; DAP: dose area product; mGy/M^2^: milligyres per meter square

Parameter	Mean	
	Phase I, N=8	Phase II, N=65
Duration (min)	36.8	31.2
Fluoroscopy time (min)	9.0	11.6
Total K (mGy)	806.2	716.2
DAP (mGy/M^2^)	46.6	49.5

Table 3 compares the cost analysis of hardware used in cath labs for diagnostic and interventional procedures in phases I and II. The analysis revealed a 28% reduction in medical items used for diagnostic and interventional cases in phase II compared to phase I.

**Table 3:** The cost analysis (in SAR) for both phases of the diagnostic and interventional cases SAR: Saudi Arabian Riyal

Item description		Diagnostic		Interventional	
		Phase I	Phase II	Phase I	Phase II
1	Stent	0	0	487.5	487.5
2	Balloon	0	0	25.1	115.8
3	Cathpack	400	410	400	400
4	Sheath	87.1	86.3	82.5	90.6
5	Guidewire	225.8		240	200
6	Catheters	460.3	488.3	441	465.7
7	PCI wire	38.7	0	420	362.7
8	Fenestrated/utility drapes	17.4	9.5	20	10.8
9	Manifold	0	0	0	1.5
10	Indeflator	22.7	0	153	134.6
11	Export catheter	15.7	0	0	20.3
12	Pressure wire	290.3	75	600	0
13	IVUS	508.1	393.8	393.7	492.2
14	Microcatheter	129	0	0	83.3
15	Guideliner	229.8	0	0	0
16	Y-connector	4.8	1.9	7.5	0
17	Transducer	183.9	180.5	171	182.1
18	Transradial band	35.9	36.1	41.3	37.8
Average cost per procedure (SAR)		2650	1901.04	3843	3085

Qualitative results

The framework by Braun and Clarke and six-phase guide were used to evaluate participant interviews [9]. The findings are presented in Table 4.

**Table 4:** Themes and codes of the participants' interviews

Theme	Awareness	Desire for change	Reinforcement of change	Collaborative work
Codes	No available standards or benchmarks	Ready to change	Establish benchmark	Not available for non-clinical indicators
	Courses	Opportunity for personal improvement	Immediate feedback	Regular cath lab meetings
	Prices are not known	Focus on inventory or speed can jeopardize patient safety	Motivation	Conference room near cath lab
	Training	Pre-cath time should be the focus	Monthly reports	Visit international centers
	Non-clinical indicators are not a priority	Improving the cath lab	Alert system	Writing a policy
		Meet international standards	Counseling	Celebrate the success

## Discussion

Quantitative results

The phase I total K study findings showed that the safety variable and radiation dose for diagnostic and interventional cases were comparable to those reported in a previous study [10]. The reduction was more significant in diagnostic cases (52%) than in interventional cases (11%), which can be explained by the solid standard performance approach in diagnostic cases. These findings were consistent with the results of similar studies conducted by Kuon et al. [7] and Patel et al. [11].

The current study found that a 27% reduction in speed was noticeable in diagnostic cases, compared to 15% in interventional cases. However, no existing research has compared the length of the procedure itself. During phase II, it was observed that the cost reduction (inventory usage) was more prominent in diagnostic cases, up to 28%, compared to interventional cases, in which the reduction was 11%. This provides evidence that leadership interventions can reduce inventory usage, as suggested by Blankenship et al. [12]. The quantitative findings from this study show that internal benchmarking can aid the change process and enhance the safety and efficiency of PSCCQ's cath lab operations. This is based on recommendations made by the Society of Cardiovascular Angiography and Interventions [13]. On the other hand, while benchmarking was not the study's primary objective, the encouraging results showed the need for multicenter benchmark studies of cardiac centers in Saudi Arabia.

Qualitative results

All of the participants displayed great interest and positivity in the proposed changes. They fully comprehended the necessity of embracing change in the context of the Saudi 2030 vision and complying with international standards. Some even viewed the transition as an opportunity for personal growth and the development of the cath lab. However, it was noted that not all participants had the same attitude towards change. One participant expressed concern, stating that he feared that prioritizing speed and inventory might compromise the safety of the patients.

**Figure 1:**
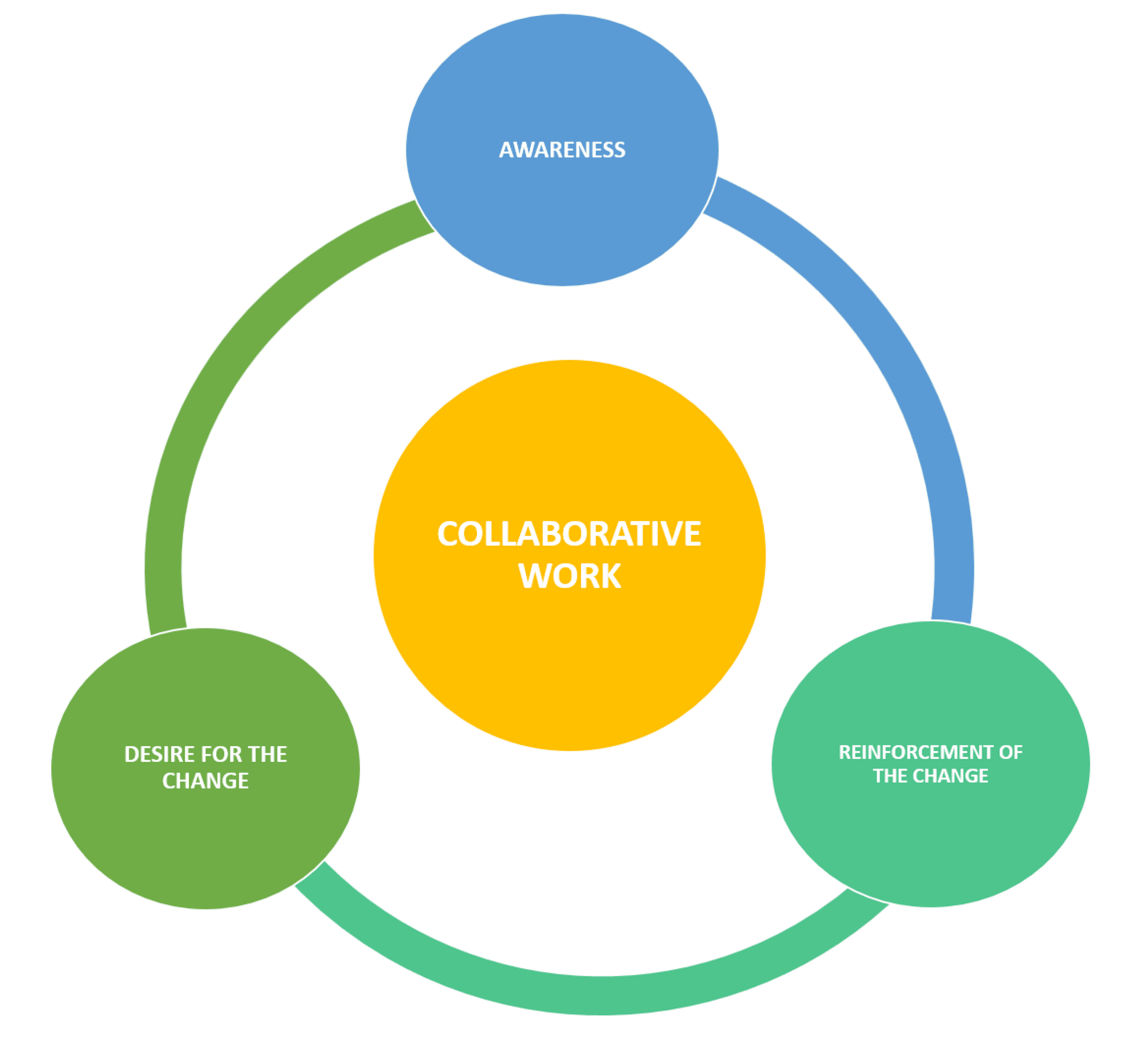
Final thematic map

This statement is critical because it could threaten the success of any leadership or change projects [14]. The present study aimed to uncover the participants' comprehension of the study variables despite needing formal training on radiation safety and cost items and benchmarks. One of the participants, who was new to the field, expressed, "My priority is to perform the procedure itself, not to worry about inventory and speed." This statement raises concerns about the need for more managerial and leadership skills training in interventional fellowship programs. All participants emphasized the need for training and education on why and how the cath lab can focus on performance indicators, including but not limited to study variables. A junior cardiologist observed that the study positively influenced many participants' behavior regarding their use of items and expressed a desire for the study to continue. It is possible that this behavioral change is a result of participants becoming more aware of their product use. Alternatively, it could be a result of the Hawthorne effect, which is the increased awareness of being observed during a study [15]. The participants reached a consensus that the emphasis on speed should be on the start-up and turnover time rather than the intervention time itself. They believed that this approach would enable more efficient utilization of the cath lab time. This suggestion was also supported by several scholars, including Blankenship et al. [12]. During the discussion, they all arrived at the consensus that reinforcement is crucial for the success of any change project. They recommended immediate feedback upon completion of a procedure, monthly written feedback, establishing benchmarks, and implementing an alert system for any deviation from the approved standards. During the study, participants were asked what they would do when a cardiologist needed to follow the guidelines. All the participants replied that the cardiologist should be informed and counseled and that they must adhere to the guidelines. This response shows that people's mindsets can change when they are made aware and their opinions are considered during the transition process. Participants believed in integrating motivation into the reinforcement process and suggested giving appreciation letters. The study found that the participants were not effectively collaborating to mitigate the radiation dose, reduce costs, and improve speed. However, they all agreed that a monthly cath lab meeting should be held and attended by the medical director or the sponsor of the change. The meeting should address clinical indicators and other important variables affecting the cath lab's performance. During a discussion, a participant suggested having a conference room near the cath lab for convenience and to encourage collaborative work. However, some of the other participants disagreed and felt that collaborative work requires a change in attitude and behavior. They recommended addressing the suggestion further at the proposed monthly meeting. The qualitative analysis of this study suggests that PSCCQ cardiologists exhibit optimistic attitudes toward change if the change leader approaches them in an understandable and inspiring manner. According to the qualitative analysis, the collaboration between the participants was not effective and difficult to achieve. The reason for this could be the diverse nationalities, cultural backgrounds, and training experiences. Although some participants suggested solutions to address this issue, the study emphasizes that a leader must put in significant efforts to improve teamwork and promote a collaborative approach. The final thematic map, as presented in Figure 1, depicts the interconnections between various themes.

Implications for leadership

The study's results show that the leadership interventions were feasible and well-received by the adult cardiologists. Additionally, these interventions have led to a safer and more effective cath lab environment for patients. Healthcare services should prioritize patients as the main motivation for change, teamwork, and innovation, especially in the face of increasing demands and limited resources.

Strengths and limitations

The study employed mixed methods to evaluate how leadership affects the performance of consultants. Three indicators (safety, speed, and cost) were used to examine the phenomenon from different perspectives, strengthening the study. However, due to the coronavirus pandemic, the study was limited, with fewer cases and a disrupted working environment. It was a challenge as an investigator, but participants were informed that the study would not be included in their annual assessment. The study demonstrates strength by closely aligning with the goals of Saudi Vision 2030 and delving into a concept that has previously received little attention in Saudi Arabia. It employed a mixed-methods approach to investigate the targeted phenomenon thoroughly. However, the coronavirus pandemic hampered the study's progress, and a longer research period would have been advantageous to mitigate the impact of this unforeseen circumstance. For future endeavors, it would be more effective to involve a change agent and conduct the study across multiple centers in various cities throughout the Kingdom of Saudi Arabia in order to obtain a comprehensive understanding of the subject matter.

## Conclusions

The current research demonstrated how benchmarking strategies can improve the performance of adult interventional cardiologists in a catheterization laboratory. Moreover, this study utilized benchmarking as a leadership tool to analyze the behavior and attitude of cardiologists in an organization toward change and collaborative practice. Quantitative and qualitative evaluations examined the same phenomenon from different perspectives. According to the research, the interventionists were willing to change the catheterization laboratory to ensure patient safety and efficacy. The interventionists successfully reduced the radiation dose in diagnostic and interventional cases, resulting in a cost reduction of 28% in diagnostic and 11% in interventional cases. The study highlights that a significant obstacle to the success of any leadership intervention is the team's need for more awareness. It also establishes that reinforcement is crucial for maintaining behavioral progress in a catheterization laboratory. However, it raises concerns about teamwork and emphasizes the need for collaborative leadership. Lastly, it underlines the need to appoint a change agent and a benchmark development officer to facilitate and sustain improvement projects in the catheterization laboratory.
